# A9 IMPLEMENTATION STRATEGIES TO OPTIMIZE DIAGNOSTIC ACCURACY OF COMPUTER-ASSISTED OPTICAL POLYP DIAGNOSIS

**DOI:** 10.1093/jcag/gwae059.009

**Published:** 2025-02-10

**Authors:** M Oleksiw, R Djinbachian, D von Renteln

**Affiliations:** Gastroenterology, Montreal University Hospital Center, Montreal, QC, Canada; Gastroenterology, Montreal University Hospital Center, Montreal, QC, Canada; Gastroenterology, Montreal University Hospital Center, Montreal, QC, Canada

## Abstract

**Background:**

Artificial intelligence (AI) has enabled the development of computer-aided diagnosis (CADx) systems which offer real-time endoscopic pathology prediction of colorectal polyps. However, the clinical benefit of CADx assisted optical diagnosis remains questionable due to lack of diagnostic ability improvement compared to non CADx assisted optical diagnosis.

**Aims:**

This study aimed to assess diagnostic performance of a novel implementation framework in which optical diagnosis replaces pathology only if CADx and endoscopist agree on polyp diagnosis. We aimed to compare this proposed framework to non CADx assisted optical diagnosis.

**Methods:**

We performed a secondary analysis of a prospective cohort undergoing optical polyp diagnosis at our center. Polyps measuring ≤5mm (diminutive) with available non CADx assisted and CADx assisted optical polyp diagnosis documentation were included in our analysis. In CADx assisted cases, first the CADx diagnostic output was documented, followed by the endoscopist’s final diagnosis after having seen the CADx diagnostic information. Only cases where the endoscopist agreed with the CADx output were retained for CADx assisted optical diagnosis. Primary outcome was the diagnostic accuracy for cases in which the endoscopist agreed with the CADx diagnostic output versus non CADx assisted OD, using pathology as a reference standard. Secondary outcomes included surveillance interval agreement, prevalence of high-confidence diagnoses, and rectosigmoid negative predictive value (NPV).

**Results:**

A total of 817 polyps were included in our analysis. Endoscopists agreed with the CADx diagnostic output in 74.3% of cases. Using CADx-assisted optical diagnosis based on CADx and endoscopist diagnostic agreement, 326/439 diminutive polyps could undergo optical polyp diagnosis in the CADx assisted arm and 378/378 in the non CADx assisted OD arm. Using diagnostic agreement between endoscopist and CADx as a framework for CADx assisted optical diagnosis demonstrated superior diagnostic accuracy, 82.8% (95% CI, 78.7-86.9), compared to accuracy of non CADx assisted optical diagnosis, 76.7% (95% CI, 71.3-80.0) (p=0.0256).

**Conclusions:**

Our study demonstrates that using cases with diagnostic agreement between endoscopist and CADx increases diagnostic accuracy for CADx-assisted OD implementation. Using this framework, CADx assisted OD outperforms non CADx assisted optical diagnosis.

**Funding Agencies:**

None

